# Exploring and Exploiting Disease Interactions from Multi-Relational Gene and Phenotype Networks

**DOI:** 10.1371/journal.pone.0022670

**Published:** 2011-07-29

**Authors:** Darcy A. Davis, Nitesh V. Chawla

**Affiliations:** Interdisciplinary Center for Network Science and Applications, Department of Computer Science and Engineering, University of Notre Dame, Notre Dame, Indiana, United States of America; Memorial Sloan Kettering Cancer Center, United States of America

## Abstract

The availability of electronic health care records is unlocking the potential for novel studies on understanding and modeling disease co-morbidities based on both phenotypic and genetic data. Moreover, the insurgence of increasingly reliable phenotypic data can aid further studies on investigating the potential genetic links among diseases. The goal is to create a feedback loop where computational tools guide and facilitate research, leading to improved biological knowledge and clinical standards, which in turn should generate better data. We build and analyze disease interaction networks based on data collected from previous genetic association studies and patient medical histories, spanning over 12 years, acquired from a regional hospital. By exploring both individual and combined interactions among these two levels of disease data, we provide novel insight into the interplay between genetics and clinical realities. Our results show a marked difference between the well defined structure of genetic relationships and the chaotic co-morbidity network, but also highlight clear interdependencies. We demonstrate the power of these dependencies by proposing a novel multi-relational link prediction method, showing that disease co-morbidity can enhance our currently limited knowledge of genetic association. Furthermore, our methods for integrated networks of diverse data are widely applicable and can provide novel advances for many problems in systems biology and personalized medicine.

## Introduction

Many diseases do not occur in isolation. Diseases with similar genetic, environmental, and lifestyle risk factors may be co-morbid in patients, or the disease products themselves may be risk factors for additional conditions. Also, many serious chronic diseases, such as cancer and diabetes, are complex diseases influenced by a combination of environment and epistasis between many genes [1]–[3]. In this way, diseases may share many distinct types of relationships with varying levels of impact for important problems such as patient risk or drug efficacy. Thus, a singular view of dependencies among diseases is not sufficient. Rather, disease mechanisms form a complex system. The underlying goal is to combine all available information and develop the most complete models of interaction between these many factors, simultaneously using information widely applicable and patient specific.

Schadt [3] suggests that diseases can be seen as emergent from a complex network of underlying molecular activity influenced by genes and environment. Indeed, complex networks are a natural way of representing any data with complicated dependency relationships. Unfortunately, most network studies and standard tools are insufficient for the task, limited to treating all relationship types equally or separate analysis of each type. Both of these approaches represent a loss of information. In this study, we use patient medical histories (phenotype data) and previously discovered disease-gene associations to construct, analyze, and compare disease-disease networks. We then take a novel approach to studying interplay between patients, diseases, and genes by merging the heterogeneous data into a multi-relational network and analyzing the structure of interaction between shared genes and clinical co-mordibidity. Finally, we demonstrate how the multi-relational structure can be applied to enhance the link prediction task of determining good targets for further gene association research.

Both gene-based [4] and patient-based [5] disease-disease networks, constructed similarly to ours, have been previously studied. These separate studies explore different questions, while our approach is to compare and combine the networks and take the composite view. In [6], Park et al. begin exploring relationships between the network links, showing that genetic association is correlated with co-morbidity and thus justifying integrated study. However, they do not take advantage of the network structure, and there are still many questions to be addressed for useful inference between the networks. Also, as our networks will show, diseases show far more co-morbidities than genetic links to other diseases, so direct inference based on shared genetic association only applies to a limited subset of co-morbidities. Park et al. acknowledge that many disease pairs share genes but are not co-morbid, and we will further show that there are far more disease co-morbidities without significant gene overlap. Our explicit integration of the networks facilitates inference based on a neighborhood of interactions, providing a richer pool of data than pairwise correlation.

Many other studies have explored integrating diverse evidence to answer biological questions, using various types of data [7]–[11]. We have already mentioned some of the limitations of simple correlation studies, particularly with respect to inference tasks. Another approach which has been used is classification using diverse evidence, such as the work on predicting gene-disease associations performed by Radivojac et al. in [9]. Classification has proven to be a good tool for many tasks, but we claim that network-based inference has certain differences that may be advantageous for biological data. Most currently available data, particularly on the molecular level, is incomplete, biased, and noisy, which corresponds to a great deal of missing or unreliable data. Classification methods must impute values for missing data to create positive and negative profiles for each decision class, which can hurt performance [12]. Most complex networks methods, including the link prediction method we will introduce, apply naturally to whatever partial information is known.

There have been a limited number of recent studies on link prediction in multi-relational networks [13]–[15]. Latent feature models have been extended for potentially overlapping multi-relational link prediction. In [14], each relation is separately predicted based on a global set of latent features by generating a separate set of weights for each feature. In [15], a single set of prediction weights is scaled by a different factor for each relation. Unlike these approaches, the main focus of our work is directly capturing the correlation structure between relationship patterns.

## Results and Discussion

### Network Descriptions

We constructed a phenotypic disease network (PDN) [5] from real patient data in which nodes are diseases and edges indicate co-morbidity of the diseases. Co-morbidity can be broadly defined as co-occurrence in the same patients significantly more than chance. We included edges between disease pairs for which the co-occurrence (joint probability) is significantly greater than the random expectation based on population prevalence of the diseases (product of marginal probabilities). Statistical significance is determined by a one-tailed two proportion z-test with 95% confidence. Additionally, diseases are required to have a minimum co-occurrence in 2 patients to avoid noise from lone rare events. Diseases with no significant relationships are omitted. For additional details about the data, see Materials and Methods. Our phenotypic disease network consists of 437 unique diseases nodes and 40,579 co-morbidity relationships, creating a very dense network.

We also constructed a genetic disease network (GDN) [4] from gene-disease associations compiled from previous studies. Nodes are unique diseases, which are connected when the diseases share a significant number of genetic associations. Similar to the PDN, disease pairs have an edge if they share significantly more gene associations than randomly expected based on the generality of the diseases. We approximated the generality with the marginal probability of the disease being associated with a random gene from the dataset. Again, significance was decided by a two proportion z-test with 95% confidence. The genetic disease network has 399 nodes connected with 7817 significant genetic links.

For the methods in this paper, we primarily utilized unweighted networks. However, we found a weighting scheme to be useful for some observations. We weighted the edges using a mutual information metric which quantifies how much greater the edge relationship is with respect to chance. For details, see Materials and Methods.

Diseases considered for inclusion in the networks were limited to those which appeared in both datasets; that is, diseases which are associated with at least one gene and occur in at least one patient. However, we do not necessarily require significant relationships in both. The overlap of the network is 399 nodes; all diseases in the GDN also had at least one significant co-morbidity. However, the PDN contains 38 additional diseases that have significant co-morbidities, have some known gene associations, but are not sufficiently genetically similar to any other diseases.

In both networks, the diseases are classified by Disease Ontology (DO) codes, which have a hierarchical structure. The structure is arranged such that a code may be a subset of other codes at many levels of generality, creating long chains of ‘is-a’ relationships. For example, *Toxic pneumonitis* is a *Pneumonia* is a *Non-neoplastic lung disorder*. Obviously, the is-a relationship is fundamentally different from other edges in the networks and should be treated as such. These links are essential to the structure of the network, so they were included but not weighted.

### Network Analysis and Comparison

**Figure 1:**
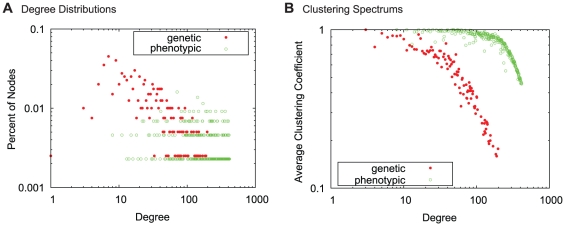
Global network properties. (**A**) Degree distributions and (**B**) clustering spectrums of the phenotypic (PDN) and genetic (GDN) disease networks. The PDN has higher average degree and clustering coefficient due to very high edge density. Interestingly, the degree distribution of the GDN generally decreasing while the PDN is more uniform, indicating that many diseases are co-morbid with a large number of other diseases, often with few or no underlying shared genes.

For each network, we calculated the degree distribution and spectrum of clustering coefficients, which are shown in Figure 1. The extremely high density of the PDN suggests that diseases generally have more co-morbidities than genetic associations. Thus, it is unsurprising that the phenotypic network has higher average degree and clustering coefficient. More interesting, however, is the remarkable difference in degree distribution. While the degree distribution in the genetic network is generally a decreasing function, the phenotypic degree distribution is more uniform. Neither of these networks have a power-law degree distribution [16]. Since these networks mostly contain the same nodes, this difference indicates that many conditions are highly co-morbid despite few or no shared genes. For example, *migraines* do not have significant genetic link to any other disease, but are co-morbid with more than 200 conditions. Note that a lack of genetic edges does not mean the conditions are not genetic, but rather that their known genetic profile is not similar to other diseases.

**Figure 2:**
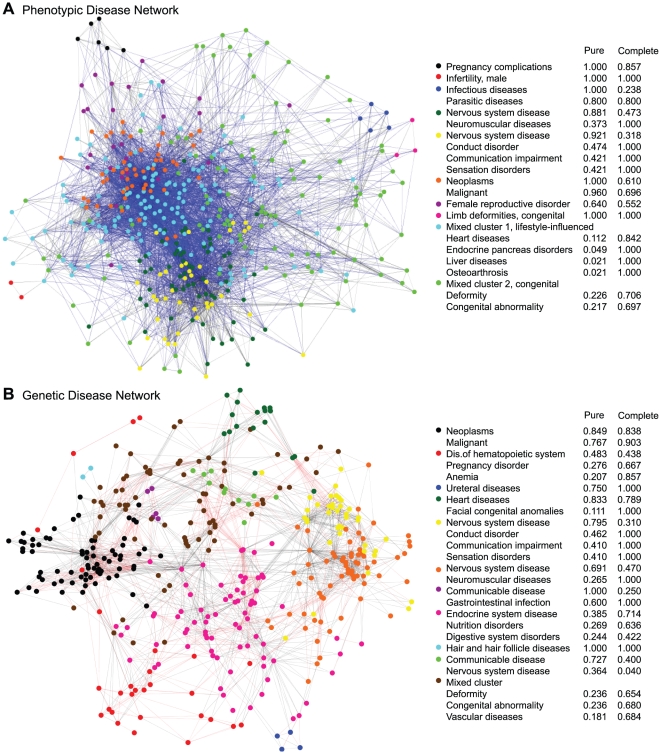
The Phenotypic and Genetic Disease Networks. (**A**) The phenotypic disease network (PDN) is constructed based on clinical history of 700,00 patients. Each node represents a unique disease, and two nodes are connected if the diseases co-morbid significantly more than randomly expected according to population prevalence. (**B**) The genetic disease network (GDN) is constructed on the same disease nodes, but edges instead indicate that the disease pair shares a significant number of gene associations. In both networks, black edges indicate hierarchically related diseases (is-a relationships). For each network, the accompanying table displays the most relevant Disease Ontology codes associated with each cluster. Purity corresponds to the percent of member nodes which are accurately described by the DO term, and completeness indicates the percentage of descendants of the DO term which belong to the cluster. For a detailed definition, see Materials and Methods. It is clear that the PDN and GDN are structurally different. Nonetheless, both networks form some easily defined clusters but also have some dense regions containing diverse DO terms.

Both networks were computationally clustered using Walktrap, a hierarchical clustering tool for networks based on the intuition that random walks are often trapped within dense network regions corresponding to clusters. Algorithm details are provided in [17]; we use the implementation provided by the authors with the default parameters. The reported clusters correspond to the partition with the highest modularity [18]. The clustered networks are provided in Figure 2, along with limited descriptions of the clusters. Due to the high density, the visual representations are limited to strongest 10% of edges in each network according to the mutual information weights. All of the nodes remain present. The reduced networks are for visual clarity only; the clusters and associated descriptions correspond to the full network. We describe the content of each cluster by finding the DO term(s) that are most pure or complete within the cluster. Each node has a DO code which is further associated with a hierarchy of more general terms. For each DO term and cluster, we define *purity* as the percentage of all cluster members which are contained by the term, and *completeness* as the percent of all nodes contained by the term that also belong to the cluster. A detailed example of these calculations can be found in Materials and Methods. Intuitively, the purity indicates the homogeneity of the cluster, while completeness measures the uniqueness relative to other clusters. Dynamic, fully labeled representations of the networks are available at http://www.nd.edu/~dial/plosone/diseasenetworks/ Cytoscape format, an open source tool for visualizing and analyzing networks [19].

The PDN was partitioned into 10 clusters, four of which are of acceptable quality due to size, purity, and completeness. By acceptable, we simply mean that the cluster is non-trivial and has a reasonably specific universal theme. These clusters can be roughly classified as *neuromuscular* and *neuro-degenerative disease*, *sensation disorders*, *malignant neoplasms*, and *female reproductive system disorders*. Four of the remaining clusters are tiny groups of related conditions, consisting almost entirely of is-a edges. The final two clusters are enormous “catch-all” clusters of mixed conditions, accounting for 56.98% of all disease nodes in combination. One of these large clusters contains the famously complex relationships between *heart disease*, *diabetes*, *strokes*, *obesity*, and many other chronic diseases believed to be lifestyle influenced. The other large cluster contains most of the *congenital deformities*. Both of these clusters additionally contain many other disease categories which were not easily separable, forming a chaotic picture of intra-connections across disease families and organ systems.

The GDN was separated into 11 clusters, with seven high quality clusters. Again, neoplasms and nervous system disease form fairly pure clusters. Genetic clusters also form for *heart disease*, *endocrine diseases*, and *diseases of the hematopoietic system*. Similar to the PDN, there are 3 tiny clusters, but only one mixed cluster of moderate size, accounting for about 19.55% of the nodes. Overall, clusters in the GDN are more specific and separated than the PDN, although in both cases there are many conditions which do not form distinct modules.

### Network Integration

The PDN and GDN provide insight into the way genes associate with diseases and the way diseases occur in patients, but little information about the interplay between the two mechanisms. The structures suggest that the two networks express different information for understanding disease mechanisms, which is not unexpected. There are multiple possible reasons, both biological and artificial, which we can speculate are behind these differences. Two diseases being associated with the same gene might not have a practical effect, especially if the diseases are associated with different loci, alleles, or expression levels. This corresponds to a connection in the GDN and none in the PDN. In the other direction, some diseases may be co-morbid only because they are influenced by the same environmental conditions. Finally, both networks are likely to have collection biases, although we expect that the PDN is more complete. However, these differences do not preclude the possibility of important patterns and dependencies between the structures. We combined the individual network information into a multi-relational disease network (MRDN) and probabilistically analyzed local structures to draw more specific conclusions about how genetic influences relate to disease co-morbidity.

We previously mentioned that the patient-based and gene-based networks were constructed from the same pool of diseases. While some diseases have significant relationships only in the PDN, the majority of the node sets overlap. Thus, the networks contain many of same disease nodes but with a different pattern of connections and weights. This allows them to be easily overlaid and represented as a single multi-relational network with multiple edge types. The edge type can be thought of as a nominal edge attribute. If both a phenotypic and a genetic edge is present between two diseases, it was treated as a single edge of type ‘both’. The multi-relational structure also allows the is-a relationship to be explicitly treated as a unique edge type. This fundamental disease relationship supersedes any other correlations. Thus, the MRDN has four possible edge types: *(G)enetic*, *(P)henotypic*, *(B)oth* genetic and phenotypic, and *(I)s-a*. The clusters from the original networks can be retained as separate node attributes.

**Figure 3:**
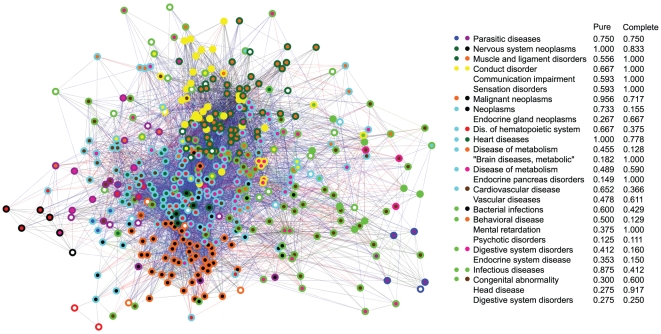
The Multi-Relational Disease Network. This network is created by overlaying the phenotypic (PDN) and genetic (GDN) networks, which contain the same disease nodes. Blue edges indicate phenotypic links, red edges are genetic, green edges are both genetic and phenotypic, and black edge are is-a relationships. The two-tone nodes indicate original cluster membership in the GDN (inner circle) and PDN (outer circle). Regions where multiple nodes share the same color pattern correspond to groups of diseases which cluster together in both the PDN and the GDN. These overlaps are common and in some cases quite large, such as the teal-and-green cluster containing the heart diseases. Still, none of the overlaps fully contain a PDN or GDN cluster. The overlapping regions are listed in the accompanying table, along with the most relevant Disease Ontology codes associated with the cluster.

**Figure 4:**
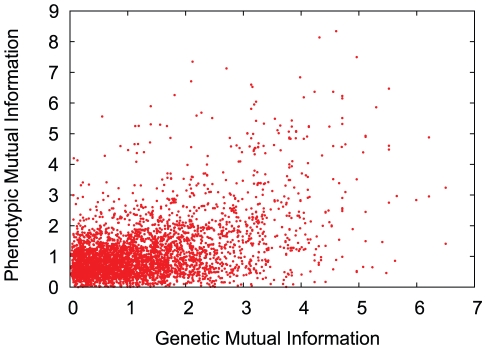
Genetic vs. phenotypic mutual information. Each data point represents a disease pair which is linked in both the PDN and the GDN. The plot illustrates the correlation between the mutual information edges weights in each respective network. There is some upward trend but the effect is far from linear. In aggregate, the values have a Pearson correlation of .473, a weak-to-moderate positive correlation.

The integrated MRDN is shown in Figure 3. The edge colors represent the relationship type. The two-tone nodes indicate original cluster membership in the GDN (inner circle) and PDN (outer circle). Areas matching the white background color indicate that the node was omitted from one of the networks. Groups of nodes with a matching two-tone pattern are overlaps between clusters found in the separate networks. It is visually apparent that substantial overlap between the cluster results is common. However, the clusters never fully overlap, nor are they contained within each other. In general, phenotypic influence tends to extend far beyond the bounds of genetic similarity. Consistent with Park et al.' s result that sharing genes is correlated with co-morbidity [6], we observe that 72% of genetic edges underlie a phenotypic edge. In Figure 4, we plot the genetic mutual information versus the phenotypic mutual information for the 4465 disease pairs which have both relationships. While the positive correlation between the values is highly significant (Pearson correlation of 0.473, probability of random observation 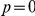 according to a Monte Carlo permutation test of 1 million permutations), it is a weak-to-moderate correlation in the general sense, i.e. the strongest genetic relationships do not necessarily translate to high co-morbidity, and vice versa. More details on the permutation test are available in the Materials and Methods Section.

Despite visually different structures, there are definite dependencies between genetic association and co-morbidity, but pair-wise correlations are weak indicators on their own. Nonetheless, even weak evidence can be very valuable for inference tasks, particularly in combination with complementary evidence, which is our approach. Furthermore, we suspect that the PDN can be very valuable for inference in regions of the GDN which have been sparsely studied, perhaps due to rarity or low morbidity.

### Multi-relational Local Structure

Construction and manual observation of the multi-relational network has already confirmed significant interplay between the genetic and phenotypic networks. However, there are still many questions about the basic rules and probabilities that govern these influences, particularly in terms of strength. In addition to furthering biological understanding of disease mechanisms, understanding the probabilistic properties of the network structure will be instrumental to locating additional genetic associations or recognizing the role of genetics in poorly understood co-morbidities.

**Figure 5:**
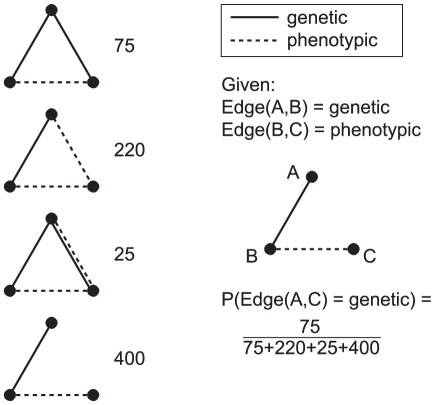
Finding edge probabilities given partial structures. This toy example demonstrates how to calculate the probability of a specific edge type closing an open triad pattern, based on the triad counts for the full network. This calculation corresponds the Equation 3. The numbers in this example do not represent the real network. The table of actual edge probabilities for the MRDN can be found in Table S1.

We approach these global questions through the local substructures, which can provide manageable and interpretable insights into the global structure [20]. For this study, we counted the occurrence of each unique 3-node structure, traditionally called triad census [21], [22] and more recently defined as counting 3-node graphlets [23]. Triad census has been widely used in social network analysis, often for evaluating local structure hypotheses such as transitivity [24]. The triad census trivially extends to multi-relational networks; the only difference is the number of unique structures. While a traditional directed network yields 16 possible structures, our network has 30 unique connected triad patterns of the four edge types. Of course, the hypothesis space becomes increasingly complex with each additional relation. For this work, our use of triad counts is more similar to the context of recent work on graphlet distribution, where substructure counts are used to thoroughly characterize the local structure [25]. Instead of hypothesizing about which structures are important, we wish to probabilistically weight all relationship patterns. The triad census provides the probability of each structure, which further translates to the probability that a partial triad is closed by each edge type. A pictorial example is shown in Figure 5, and the probabilities found in the real network can be seen in supplementary Table S1.

### Multi-Relational Link Prediction

One of the great challenges for studying biological networks and systems biology in general is the incompleteness and noise of the data. In any large scale molecular context, especially considering phenomena such as epistasis, experimentally exhausting all combinations is not a viable option. Even experimental studies, especially high-throughput methods, may be inconsistent or can result in high false positive rates, thus requiring many trials or diverse evidence to be reliable. Computational approaches such as disease-gene prioritization are essential for targeting future experiments, directing time and money towards the most likely successes.

In complex networks, finding missing associations is the link prediction problem, which can be broadly generalized as follows: Given two nodes 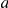 and 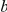 that are not connected by an edge, predict whether the edge actually does exist, or in the case of dynamic interactions, will form in the near future. Usually, this prediction is in the form of a score for each disease pair. The scores are then ranked to determine the nodes pairs that are relatively most likely to have an edge. Many link prediction methods exist for networks; a survey of these methods can be found in [26], [27]. For this work, we focus on unsupervised topological models.

However, most traditional link prediction methods have no direct applicability to multi-relational networks other than treating all edges equally, which can be detrimental to their performance for many reasons. Different link types contain different information by nature, and various combinations introduce different amounts of evidence to the link prediction task. This is particularly troublesome when the link types have very different frequency or distribution, which is clearly the case in our multi-relational disease network. In a sufficiently complicated system, some edge types may be irrelevant or redundant with respect to certain prediction tasks. Even if these barriers could be overcome, treating all edges equally provides no information about the type of link being predicted.

We propose a novel multi-relational link prediction (MRLP) method which addresses all of these issues to predict the location and type of new edges. The most important component of our MRLP method is an appropriate weighting scheme for different edge type combinations. In *Multi-relational Local Structure*, we explained how a triad census can be used to place a probability on local substructures, which conveniently translates to a non-arbitrary, data-justified weighting scheme. To account for frequency disparity, the probabilistic weights are normalized by the marginal probabilities of the edge types involved. Implementation details of our MRLP method and related traditional link predictors can be found in Materials and Methods. Ours is a general algorithm for multi-relational networks, which can also be trivially extended to multiple node types.

**Figure 6:**
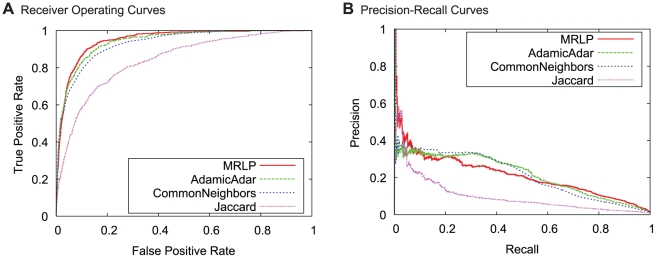
Link prediction performance. (**A**) Receiver operating curves (ROC) and (**B**) precision-recall curves for the multi-relational link predictor (MRLP) and three traditional neighborhood-based link prediction methods: common neighbors, Jaccard coefficient, and the Adamic/Adar measure. MRLP is the best method with respect to area under the receiver operating curve (AUROC). The precision-recall curve, which is less biased, shows that MRLP is most accurate with the highest ranked predictions, but is not always optimal for lower prediction thresholds.

We applied our probabilistically weighted MRLP to the multi-relational disease network. For this application, we only generated prediction scores for genetic links, since ‘is-a’ relationships are known and we assume that the patient data completely represents significant co-morbidities. We compared our performance to traditional neighborhood-based link prediction methods as applied to the genetic disease network (GDN). The algorithms used are Common Neighbors, Jaccard coefficient, and the Adamic/Adar measure (details in Materials and Methods). These methods provide a baseline for how well the genetic links in our network can be predicted without the benefit of multi-relational analysis. For all experiments, we use 10-fold cross validation, holding out 10% of the genetic edges for each run. The comparative performance is shown by the receiver operating characteristic (ROC) curves and precision-recall curve [28] in Figure 6. The MRLP outperforms the traditional methods with respect to AUROC. The precision-recall curve, which is potentially less biased by the extreme imbalance between actual links versus all possible pairs, shows that MRLP performs particularly well on the top 50 rankings, but is not optimal for all decision boundaries, as one would expect. The drop in precision as recall increases is expected, as it is accompanied by an increase in false positives. When predicting possible genetic links for further investigation, it is also important to have fewer false positives, and thus the operating range might be constrained to top-50 or top-100 rankings (precision at 50 or at 100, for instance). The MRLP also reaches 100% recall with higher precision than the other methods, which we hypothesize is due to an improved ability to distinguish between edges with very low genetic evidence. These results indicate that phenotypic information can help improve the prediction of genetic links between diseases, even though less than 12% of the phenotypic relationships coincide with an underlying genetic association.

**Figure 7:**
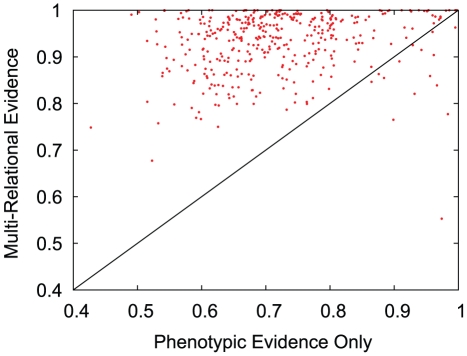
Link predictor performance by individual disease. Area under the receiver operating curve (AUROC) comparison of link predictor performance for each unique disease. The experiments were hold-one-out, where all genetic associations of the testing disease were removed. The x axis shows the performance of Adamic/Adar on the phenotypic data only, and the y axis is the performance using the MRLP on the multi-relational network. Each point which falls above the diagonal indicates that multi-relational evidence improved link prediction performance for the corresponding disease.

We then applied the MRLP to a more difficult problem: a disease with no known genetic associations, only a phenotypic profile. Such a disease is disconnected in the GDN, and thus cannot be predicted by the baseline algorithms applied to the GDN as in previous experiment. The link predictions can be made based on the PDN, but phenotypic evidence alone is weak. The multi-relational approach provides a connection while allowing the genetic associations of the other diseases in the network to still play a role. Experimentally, we simulated this scenario by holding out all genetic associations for each disease individually, and then using the MRLP to predict the correct locations of the removed associations. Figure 7 shows the AUROC achieved for each disease using the MRLP versus Adamic/Adar applied to phenotypes only. Similar trends hold for MRLP versus the other benchmark algorithms, slightly shifted leftward due to lower average performance. The strong majority of diseases fall above the diagonal, indicating that the multi-relational approach improved the predictions for that disease. The genetic associations were most easily predicted using phenotypic relationships for *alopecia*, *hypothyroidism*, and *complications of diabetes mellitus*. The most poorly predicted were *schizophrenia*, *polymyositis*, and *frontotemporal dementia*.

## Materials and Methods

### Ethics Statement

The IRB at the University of Notre Dame, deemed the research to be Exempt Under Category 4 (Research involving the collection or study of existing data, documents, records, pathological specimens, or diagnostic specimens, if these sources are publicly available or if the information is recorded by the investigator in such a manner that subjects cannot be identified, directly or through identifiers linked to the subjects.).

The assurance number at Notre Dame is FWA00002462 expiration 09/23/2013 and IRB NUMBER is 00000329.

The exempt document is filed in records, and is dated 18th March, 2009.

### Data

We determined genes shared between diseases based on known disease-gene associations extracted from OMIM, Swiss-Prot, and HPRD. The diseases are classified by Disease Ontology (DO) codes and the gene names are based on the HUGO Gene Nomenclature. The Disease Ontology project is intended to develop a controlled medical vocabulary to unify diverse medical languages and ontologies such as UMLS, ICD, and SNOMED. It is implemented as a directed acyclic graph indicating the hierarchical structure of the disease terms.

Disease co-morbidity was calculated from real patient medical histories collected from a group of 77 physicians within a regional health system. This includes data for the last 12 years, from 1997 to 2009, with a total of 5.5 million visits for approximately 700,000 patients. Each data record is a single visit represented by an anonymized patient ID and a primary diagnosis code, as defined by the International Classification of Diseases, Ninth Revision, Clinical Modification (ICD-9-CM). For consistency with the first dataset, the ICD-9-CM codes have been converted to Disease Ontology codes based on mappings provided within the DO coding. The mapping is many to many, so a single ICD-9-CM code often translates to a list of DO codes, and a DO code may apply to multiple ICD-9-CM codes as well.

### Mutual Information Weighting

The mutual information weight 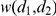 between two diseases 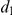 and 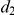 is defined as
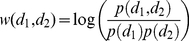
(1)where the numerator is the observed co-occurrence (joint probability) and the denominator is the random expectation of co-occurrence (product of marginal probabilities). Co-occurrence refers to the number of shared patients in the PDN, or the number of shared genes in the GDN. This weighting scheme is used to avoid bias based on disease prevalence, which is necessary since previous work with the data [29] has shown that a few common diseases tend to dominate other correlations.

### Permutation Test

In order to determine if genetic mutual information and phenotypic mutual information are significantly correlated for the disease pairs in our networks, we used a Monte Carlo permutation test. Each permutation was determined by randomly pairing each genetic mutual information value with a phenotypic value using a Fisher-Yates shuffle of each value set, respectively. We generated 1 million permutations, each with a corresponding Pearson correlation value. The correlation values were within the range 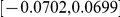 with a mean value of 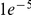. The observed Pearson correlation in our networks was 0.473, which falls well outside the range generated from the permutations, which corresponds to probability 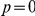 of random observation. We concluded with high confidence that genetic and phenotypic mutual information of the disease pairs is significantly positively correlated.

### Cluster Purity and Completeness

As mentioned in *Network Analysis and Comparison*, we calculate the purity and completeness of each network cluster with respect to each DO term associated with the cluster members. We defined *purity* as the percent of all cluster members which belong to a given term and *completeness* as the percent of all nodes belonging to the term that also belong to the cluster.

We now provide a detailed example of this calculation for a cluster of disease terms {*coccidiosis, malaria, arthropod diseases, helmintiasis, parasitic intestinal diseases*}, which corresponds to the five blue nodes on the upper right side of Figure 2A. These are all children of the DO term *infectious diseases*, so the purity with respect to that term is 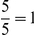. However, these are only 5 of the 21 *infectious diseases* in the network, so the completeness is 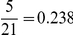. Similarly, 4 of the 5 terms are *parasitic diseases* (purity: 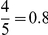) and only one other parasitic disease is included elsewhere in the network (completeness: 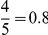).

Purity and completeness can be determined for all cluster-DO<1?tlb 10.5pt?> term pairs. The terms provided in Figure 2 are those that best describe each cluster. While the determination of the best terms was subjective, most clusters had clear winners. Otherwise, the clusters have been marked as “mixed”.

### Link Prediction Algorithm

Our multi-relational link prediction (MRLP) approach works as follows. Nodes 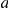 and 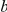 form a partial triad with each common neighbor, and each partial triad provides a probabilistic weight based on the triad census. We can then add the weights. Prediction scores are found individually for each link type of interest. Formally, the prediction score for edge type 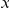 between nodes 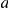 and 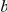 is
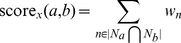
(2)where
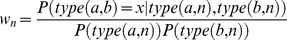
(3)


As mentioned earlier, the denominator of the weight term is a normalization factor to account for the frequency disparity between edge types.

We further extend this equation to include the inverse frequency principle, since it has been shown to increase performance in many cases. The integration is direct except that the degree is counted only with respect to the relevant node types. The prediction score becomes
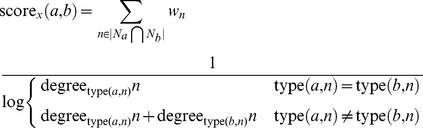
(4)where 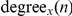 is defined as the number of edges of 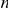 with edge type 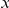.

In supplementary Text S1, we describe additional approaches using hierarchical clustering information as prior weighting for the MRLP, which did not prove to be beneficial.

#### Benchmark Methods

We considered the neighborhood-based topological methods, which are better suited to our networks and make for a strong benchmark to the proposed MRLP. In this case, topological refers to methods that rely only on the structure of the network to draw conclusions.

*Common neighbors* is the simplest method, where the link prediction score for the node pair 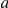 and 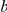 is
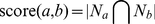
(5)where 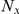 is the set of all nodes connecting to node 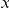 (the neighbors of 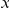). Another well-known common neighbor method is the *Jaccard* coefficient, where
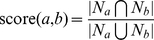
(6)


A third variation which usually improves performance significantly is the *Adamic/Adar* measure [30], which weights the impact of neighbor nodes inversely with respect to their total number of connections. Specifically,
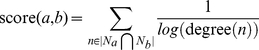
(7)


This inverse frequency approach is based on the principle that rare relationships are more specific and have more impact on similarity, which is justified in our network (recall the clustering spectrum, Figure 1) and many other real world scenarios.

## Supporting Information

Table S1 Triad probabilities derived from the multi-relational disease network.
(PDF)

Text S1 An experimental approach for including Disease Ontology distance and hierarchical clustering weights in MRLP.
(PDF)
