# Supplementary material for: Exploring and Exploiting Disease Interactions from Multi-Relational Gene and Phenotype Networks
Source: PLoS One. 2011 Jul 29;6(7):e22670. doi: 10.1371/journal.pone.0022670 (PMC3146471; doi:10.1371/journal.pone.0022670)
Supplement: Table S1 — Triad probabilities derived from the multi-relational disease network. (PDF) [file pone.0022670.s001.pdf]

N - no edge	P(N—I,I)=0.433	P(N—I,P)=0.363	P(N—G,G)=0.438	P(N—G,B)=0.347	P(N—P,B)=0.283
I - is-a edge	P(I—I,I)=0.240	P(I—I,P)=0.021	P(I—G,G)=0.074	P(I—G,B)=0.065	P(I—P,B)=0.028
G - genetic edge	P(G—I,I)=0.034	P(G—I,P)=0.006	P(G—G,G)=0.169	P(G—G,B)=0.103	P(G—P,B)=0.016
P - phenotypic edge	P(P—I,I)=0.184	P(P—I,P)=0.533	P(P—G,G)=0.174	P(P—G,B)=0.282	P(P—P,B)=0.567
B - both G and P	P(B—I,I)=0.110	P(B—I,P)=0.077	P(B—G,G)=0.146	P(B—G,B)=0.204	P(B—P,B)=0.107
P(N)=.556	P(N—I,G)=0.468	P(N—I,B)=0.366	P(N—G,P)=0.472	P(N—P,P)=0.325	P(N—B,B)=0.228
P(I)=.020	P(I—I,G)=0.074	P(I—I,B)=0.054	P(I—G,P)=0.009	P(I—P,P)=0.024	P(I—B,B)=0.065
P(G)=.018	P(G—I,G)=0.156	P(G—I,B)=0.044	P(G—G,P)=0.030	P(G—P,P)=0.012	P(G—B,B)=0.046
P(P)=.359	P(P—I,G)=0.110	P(P—I,B)=0.337	P(P—G,P)=0.422	P(P—P,P)=0.567	P(P—B,B)=0.429
p(B)=.047	P(B—I,G)=0.192	P(B—I,B)=0.199	P(B—G,P)=0.068	P(B—P,P)=0.072	P(B—B,B)=0.236
