# Supplementary material for: Exploring and Exploiting Disease Interactions from Multi-Relational Gene and Phenotype Networks
Source: PLoS One. 2011 Jul 29;6(7):e22670. doi: 10.1371/journal.pone.0022670 (PMC3146471; doi:10.1371/journal.pone.0022670)
Supplement: Text S1 — An experimental approach for including Disease Ontology distance and hierarchical clustering weights in MRLP. (PDF) [file pone.0022670.s002.pdf]

Introducing Disease Ontology and Clustering Priors to Multi-Relational Link Prediction

Darcy Davis and Nitesh V. Chawla

1 Introduction

This work is supplementary material to paper *Exploring and Exploiting Disease Interactions from Multi-relational Gene and Phenotype Networks*, in which we introduce a multi-relational link prediction (MRLP) method for networks with multiple edge types. This material assumes that the reader has already read the parent paper and is familiar with our disease networks, link prediction, and the MRLP method. Also, a basic understanding of hierarchical clustering (we use Walktrap [1]) is assumed.

2 Introducing Prior Scores to the MRLP

For the experiments in this document, we assume that each pair of nodes a and b has a prior prediction score $\text{prior}(a, b)$ independent of the MRLP score. We then incorporate the prior into the final link prediction score and determine the effect on performance. We try two different methods for incorporating the priors. The first is a multiplicative prior, which is simply

$$\text{score}(a, b) = \text{prior}(a, b) * \text{MRLP\_score}$$

The second method is a more fine-grained additive prior, calculated by

$$\text{score}(a, b) = \sigma\text{prior}(a, b) + \text{MRLP\_score}$$

where σ is a scaling factor which determines the prior's strength of influence. The additive approach is sensitive to the scale of scores, so we normalize both the priors and the MRLP prediction scores to the range [0,1]. To determine the value of σ, we use a 10% holdout validation set in addition to the 10% testing set. After choosing σ, we reintroduce the validation set to the network before calculating MRLP scores for the test pairs. This avoids performance loss caused by removal of the validation set.

3 Disease Ontology Priors

The diseases in our phenotypic (PDN) and genetic (GDN) disease networks are all contained within the Disease Ontology (DO), which is implemented as a fully

connected directed acyclic graph. Ignoring the direction of edges, there is at least one path in the DO between every pair of diseases. We will refer to the length of the minimum path between two nodes as the *ontological distance*. In general, the ontological distance provides some information about the relatedness of the disease. For example, all nodes with a distance of 1 have a direct parent-child relationship; one disease is a sub-classification of the other. We hypothesize that node pairs with shorter distance in the DO are more likely to be biologically similar, and thus more likely to share associated genes. It logically follows that the ontological distance could be valuable for predicting genetic association. We used the inverted minimum distance between disease pairs as ontological link prediction weights. Specifically, the weight for a diseases is their ontological distance divided by the DO diameter.

We found that the DO weights negatively impacted the performance of MRLP when introducted as prior, for both the multiplicative and additive cases. We weren't surprised by this result. The Disease Ontology is valuable for high level observations, but is not intended for mathematical precision. Also, the minimum distance within the DO hierarchy is already represented within the networks, and is already directly used by the MRLP if the distance is 2. Greater distances could be much more elegantly implemented by simply allowing MRLP to consider larger graphlets, which is future work for us.

4 Hierarchical Clustering Priors

In many hierarchical clustering methods, including the Walktrap method that we used for the disease networks, all nodes begin in separate clusters and clusters are gradually merged until the network is whole again. For most studies, the goal is to find the best clustering based on some performance criterion. However, for the purpose of this work, we are interested in the order in which clusters are merged. In the hierarchical process we just described, all node pairs are eventually in the same cluster at some level of the tree. We hypothesize that disease which reach the same cluster earlier are more likely to have a link. We define each merging of two clusters to be a *hierarchical level*, where the first two nodes are merged at level 1. We further define the hierarchical level where two nodes first belong to the same cluster as the *introduction level*. To calculate a clustering link prediction weight for a disease pair, we use their inverted introduction level. Specifically, the weight for two diseases is their introduction level divided by the maximum level.

Each disease pair has two clustering priors from the PDN and the GDN, respectively. We introduce them both individually and simultaneously. However, in all experimental cases the priors did not result in performance gains. In this case, we suspect that using clustering information was somewhat redundant, since the principles governing clustering (dense connections between nodes) are similar to those used by MRLP and all of the neighbor based clustering methods.

References

[1] P. Pons and M. Latapy. Computing communities in large networks using random walks. *J. Graph Alg. App.*, pages 191–218, 2006.
